# SHP2 promotes proliferation of breast cancer cells through regulating Cyclin D1 stability *via* the PI3K/AKT/GSK3β signaling pathway

**DOI:** 10.20892/j.issn.2095-3941.2020.0056

**Published:** 2020-08-15

**Authors:** Yue Yuan, Yanling Fan, Zicong Gao, Xuan Sun, He Zhang, Zhiyong Wang, Yanfen Cui, Weijie Song, Zhaosong Wang, Fei Zhang, Ruifang Niu

**Affiliations:** ^1^Department of Public Laboratory, Tianjin Medical University Cancer Institute and Hospital, National Clinical Research Center for Cancer, Tianjin 300060, China; Key Laboratory of Cancer Prevention and Therapy of Tianjin, Tianjin’s Clinical Research Center for Cancer, Tianjin 300060, China; Key Laboratory of Breast Cancer Prevention and Therapy, Tianjin Medical University, Ministry of Education, Tianjin 300060, China

**Keywords:** SHP2, breast cancer, proliferation, Cyclin D1, GSK3β, PI3K/AKT

## Abstract

**Objective:** The tyrosine phosphatase SHP2 has a dual role in cancer initiation and progression in a tissue type-dependent manner. Several studies have linked SHP2 to the aggressive behavior of breast cancer cells and poorer outcomes in people with cancer. Nevertheless, the mechanistic details of how SHP2 promotes breast cancer progression remain largely undefined.

**Methods:** The relationship between SHP2 expression and the prognosis of patients with breast cancer was investigated by using the TCGA and GEO databases. The expression of SHP2 in breast cancer tissues was analyzed by immunohistochemistry. CRISPR/Cas9 technology was used to generate SHP2-knockout breast cancer cells. Cell-counting kit-8, colony formation, cell cycle, and EdU incorporation assays, as well as a tumor xenograft model were used to examine the function of SHP2 in breast cancer proliferation. Quantitative RT-PCR, western blotting, immunofluorescence staining, and ubiquitination assays were used to explore the molecular mechanism through which SHP2 regulates breast cancer proliferation.

**Results:** High SHP2 expression is correlated with poor prognosis in patients with breast cancer. SHP2 is required for the proliferation of breast cancer cells *in vitro* and tumor growth *in vivo* through regulation of Cyclin D1 abundance, thereby accelerating cell cycle progression. Notably, SHP2 modulates the ubiquitin–proteasome-dependent degradation of Cyclin D1 *via* the PI3K/AKT/GSK3β signaling pathway. SHP2 knockout attenuates the activation of PI3K/AKT signaling and causes the dephosphorylation and resultant activation of GSK3β. GSK3β then mediates phosphorylation of Cyclin D1 at threonine 286, thereby promoting the translocation of Cyclin D1 from the nucleus to the cytoplasm and facilitating Cyclin D1 degradation through the ubiquitin–proteasome system.

**Conclusions:** Our study uncovered the mechanism through which SHP2 regulates breast cancer proliferation. SHP2 may therefore potentially serve as a therapeutic target for breast cancer.

## Introduction

Breast cancer remains the most common malignant disease and the second leading cause of death due to cancer among women worldwide^1^. Despite the wide use of novel aromatase inhibitors and anti-HER2 targeting drugs to treat this disease, the long-term survival of patients with breast cancer remains unsatisfactory, owing to adaptive resistance to these therapeutic strategies^1–3^. The dysregulation of multiple oncogenic pathways leads to cancer cell survival, loss of response to drugs, and abnormal proliferation, invasion, and metastasis, all of which ultimately exacerbate the poor prognosis^4,5^. The lack of effective therapeutic targets is the main obstacle in improving clinical outcomes in breast cancer. Therefore, screening and identifying new therapeutic targets for breast cancer is urgently needed.

The Src homology 2-containing phosphatase 2 (SHP2) is a member of the nonreceptor type protein tyrosine phosphatase (PTPN) family^6^. SHP2 was initially identified as a platelet-derived growth factor receptor (PDGFR) beta binding protein^7^. Interestingly, SHP2, as a tyrosine phosphatase, is phosphorylated at tyrosine residues in the C-terminal domain by PDGFR in response to PDGF stimulation^7,8^. Phosphorylated SHP2 links PDGFR to Grab2 and positively regulates RAS activation by PDGFR^7,9^. SHP2 is a ubiquitously expressed multifunctional protein^10^. SHP2 can act as an adaptor or phosphatase and consequently regulate various cellular activities, such as migration, proliferation, survival, and apoptosis^11,12^. SHP2 is considered a key node in various growth factor, hormone, and cytokine-induced signaling pathways^13–15^. Mutations in SHP2 have been found to be responsible for certain hereditary diseases, such as Noonan and LEOPARD syndromes^10,16–18^. SHP2 mutations are associated with the pathogenesis of leukemia^19–21^ but are uncommon in solid tumors^22^. Therefore, the precise mechanism underlying how SHP2 contributes to the development of these diseases is complex and worthy of exploration.

Unlike other phosphatases that function as potential suppressors in carcinogenesis, SHP2 can act as either an oncogene or a tumor suppressor^6^. For example, SHP2 knockout in hepatocytes leads to STAT3 activation and promotes the development of liver cancer^23^. Likewise, SHP2 negatively regulates STAT3 phosphorylation and inhibits proliferation in esophageal squamous cell cancer^24^. In addition, SHP2 deletion results in the activation of hedgehog signaling and causes cartilage tumors^25^. This evidence supports the tumor-suppressing role of SHP2. In contrast, SHP2 is upregulated in breast cancer tissues, and elevated SHP2 promotes the invasion and metastasis of breast cancer cells *in vitro* and *in vivo*^14,26–28^. SHP2 also enhances the metastasis of glioma, prostate, lung, and pancreatic cancers by promoting epithelial-to-mesenchymal transition (EMT)^29–33^. The inhibition of SHP2 blocks cell growth and increases sensitivity to inhibitors of epidermal growth factor receptor and anaplastic lymphoma kinase in non-small-cell lung cancer^32,34^. These data suggest that SHP2 functions as a tumor promoter in these carcinomas. Hence, the dual role of SHP2 in tumorigenesis may be dependent on tissue type.

We previously reported that SHP2 is required for IL-6-induced EMT in breast cancer cells^35^, but whether SHP2 contributes to the proliferation of breast cancer cells, and the related mechanistic details, remained to be determined. In the present study, we demonstrate that SHP2 modulates the proliferation of breast cancer cells *in vitro* and tumor growth *in vivo* by regulating Cyclin D1 expression and thereby accelerating cell cycle progression. In support of this finding, SHP2 expression in breast cancer tissues was found to be positively correlated with tumor size and the proliferation marker Ki67. Investigation of the underlying mechanism revealed that SHP2 modulates the ubiquitin–proteasome-dependent degradation of Cyclin D1 *via* the PI3K/AKT/GSK3β/Cyclin D1 signaling pathway. These findings extend understanding of the function of SHP2 in breast cancer progression.

## Materials and methods

### Cell culture

HEK-293T and 2 human breast cancer cell lines (MDA-MB-231 and T47D) were obtained from the American Type Culture Collection (Manassas, VA, USA). MDA-MB-231 and T47D cells were cultured in RPMI-1640 medium (Hyclone, Logan, UT, USA) containing 10% fetal bovine serum (Gibco, Australia). HEK-293T cells were maintained in Dulbecco’s modified Eagle’s medium/high glucose (Hyclone, Logan, UT, USA) with 10% fetal bovine serum at 37 °C under 5% CO_2_.

### Antibodies, reagents, and drugs

CHIR99021 and PD98059 were obtained from MedChem Express (Monmouth Junction, NJ, USA). MG132 and LY294002 were purchased from Selleckchem (Houston, TX, USA). TRIzol reagent and Protein A/G agarose beads were obtained from Invitrogen (Carlsbad, CA, USA). A CCK-8 kit was purchased from Dojindo (Kumamoto, Japan). Primary antibodies against SHP2 (sc-7384), GAPDH (sc-47724), ubiquitin (sc-8017), and Cyclin E1 (sc-247) were purchased from Santa Cruz Biotechnology (Santa Cruz, CA, USA). Antibody against Cyclin D1 (ab134175) was purchased from Abcam (Cambridge, MA, USA). Cycloheximide (CHX) (#2112s) and antibodies against phospho-Cyclin D1 (T286) (#3300), total GSK-3β (#12456), phospho-GSK3β (Ser9) (#5558), Cyclin B1 (#12231s), β-catenin (#8480), AKT (#9272), phospho-AKT (T308) (#4056s), ERK1/2 (#4695), phospho-ERK1/2 (T202/Y204) (#4370s), Rb (#9309), and phospho-Rb (Ser780) (#9307) were purchased from Cell Signaling Technology (Beverly, MA, USA). Mouse monoclonal antibodies against β-actin were purchased from Sigma-Aldrich (St. Louis, MO, USA).

### Data sets

The Cancer Genome Atlas (TCGA) mRNA expression data [mRNA fragments per kilobase transcript per million mapped reads (FPKM)] and matched clinical metadata were downloaded from the Genomic Data Commons data portal (https://portal.gdc.cancer.gov/). The GSE21653, GSE2034, and GSE20685 datasets were downloaded from GEO (https://www.ncbi.nlm.nih.gov/geo). For GEO data, the PTPN11 expression value (probe: 212610_at) and clinical information in each dataset were extracted with Kaplan–Meier plotter (https://kmplot.com/). For TCGA data, the FPKM data were first transformed into transcripts per million data for better comparison, and then the PTPN11 expression value was extracted directly. The patients in all datasets were grouped into high- and low-expression groups on the basis of the median expression of PTPN11, and survival analysis was performed with the survival package in R (version 3.5.1).

### Establishment of a SHP2 stable knockout cell line with CRISPR/Cas9

SHP2-knockout breast cancer cell lines were established with CRISPR/Cas9 gene editing technology. Briefly, 2 sgRNAs (sgRNA#1: CACCGGAGACTTCACACTTTCCGTT targeting exon2 and sgRNA#2: CACCGGTTACTGACCTTTCAGAGGT targeting exon3) were designed to target the coding region of the *PTPN11* gene, which encodes the protein SHP2. The forward and reverse sgRNA oligonucleotides were synthesized, annealed, and cloned into the pLenti-Guide-Puro vector *via* the restriction sites *Bsm*BI and *Sca*I. MDA-MB-231 and T47D cells were cotransfected with pLentiGuide-sgRNA#1/sgRNA#2 and Lenti-Cas9-GFP with Lipofectamine 2000 reagent. Then GFP-positive cells were sorted into 96-well plates with a flow cytometer. The screening of single colon knockout cell lines was achieved through western blotting and Sanger sequencing. The online program Inference of CRISPR Edits (https://ice.synthego.com) was used to determine the precise genotype of the CRISPR/Cas9-edited cells.

### Viral packaging and infection

The lentiviral plasmid pCDH-SHP2 was obtained in our previous study^36^. In brief, full-length SHP2 was amplified by PCR with the following primers: upper, 5′-CGGAATTCATGACATCGCGGAGATGGTTTC-3′ and lower, 5′-GAGGATCCTCATCTGAAACTTTTCTGCTGT-3′. Then the SHP2-coding region was cloned into the lentiviral vector pCDH-CMV-MCS-EF1-Puro (pCDH) *via* the *Bam*HI and *Eco*RI cloning sites. Viruses were produced by cotransfection of HEK-293T cells with lentiviral and packaging vectors with Lipofectamine 2000 reagent. After infection with virus for 24 h, stable cell lines were selected with 2 µg/mL puromycin (Sigma, St. Louis, MO, USA).

### Western blot and ubiquitination assays

Western blot assays were performed as described previously^36^. In brief, the cells were lysed with 1× sodium dodecyl sulfate (SDS) lysis buffer, and the protein samples (40 µg/lane) were used for SDS–polyacrylamide gel electrophoresis separation and were subsequently transferred to polyvinylidene difluoride membranes. Afterward, the membranes were blocked with 5% skim milk for 1 h at room temperature and then probed with primary antibodies overnight at 4 °C. After being washed with 1× Tris buffered saline with Tween for 30 min, the membrane was incubated with horseradish peroxidase-linked secondary antibodies, and the bands were detected with an enhanced chemiluminescence kit (Millipore; Billerica, MA, USA). β-actin and GAPDH were used as loading controls. For Cyclin D1 ubiquitination assays, the cells were incubated with 10 µM MG132 for 6 h. Then the cells were washed twice with ice-cold phosphate-buffered saline (PBS), and lysed in buffer containing 20 mM Tris-HCl (pH 7.4), 100 mM NaCl, 1.5 mM MgCl_2_, 1% Triton X-100, and protease inhibitor cocktail. Cell lysates were immunoprecipitated with Cyclin D1 and 20 µL Protein G agarose beads overnight at 4 °C. Then the enriched protein samples were separated through SDS–polyacrylamide gel electrophoresis and subjected to western blotting with anti-Cyclin D1 and anti-ubiquitin antibodies.

### Quantitative reverse transcription (RT)-PCR assays

Quantitative RT-PCR assays were performed as described previously^37^. In brief, 1 µg of total RNA was reverse-transcribed into cDNA with a Moloney murine leukemia virus RT kit according to the manufacturer’s protocols. Quantitative RT-PCR analysis was performed with a SYBR premix Ex *Taq* kit. β-actin was used as an internal reference gene to normalize mRNA levels. Data were analyzed with the 2^−ΔΔCt^ method. The sequences of primers used in this study are provided in **Table 1**.

### Immunofluorescence staining

Cells were seeded at a density of 2 × 10^4^ cells/well in a 12-well plate, cultured for 48 h, and pretreated with the corresponding inhibitors for 6 h or incubated without pretreatment. Afterward, the cells were washed with PBS, fixed with paraformaldehyde, permeabilized with methanol, and counterstained with 4′,6-diamidino-2-phenylindole to visualize the nuclei. The cell images were obtained with a fluorescence microscope (EVOS; Life Technologies, Carlsbad, CA, USA).

### Cell proliferation assays

The CCK-8 and colony formation assays were used to evaluate cell proliferation ability. For the CCK-8 assays, cells were plated at a density of 1 × 10^3^ cells/well in 96-well plates. At the indicated time points, CCK-8 solution (10 µL) was added into the culture medium, and the cells were further cultured for 4 h at 37 °C. Absorbance was measured at 450 nm to determine cell viability. Three independent experiments were performed. For the colony formation assays, cells were plated at a density of 500 cells/well in 6-well plates. After 2 weeks, the cells were washed with PBS, fixed with methanol for 10 min, and stained with 0.1% crystal violet solution for 15 min. The plates were air-dried, and visible colonies were counted. Experiments were repeated 3 times in triplicate.

### Cell cycle analysis

Cells were fixed with 75% ethanol at 4 °C overnight. The cells were then centrifuged, washed with PBS, and stained with propidium iodide (50 µg/mL) in the presence of RNase A for 30 min at 37 °C. A total of 30,000 cells were analyzed with a FACScan flow cytometer (BD, Franklin Lakes, NJ, USA).

### EdU incorporation assays

A Cell-Light EdU Apollo 488 *in vitro* kit (C10310-1, RiboBio, Guangzhou, China) was used for 5-ethnyl-2 deoxyuridine (EdU) incorporation assays. In brief, the cells were plated onto a 24-well plate 1 day before the assay. Then EdU (50 µM) was added into each well and incubated for 4 h. Afterward, the cells were fixed, washed, and incubated with 2 mg/mL glycine, then permeabilized with 0.5% Triton X-100/PBS for 10 min. Finally, the cells were stained according to the manufacturer’s instructions.

### Tissue specimens and IHC

A total of 101 paraffin-embedded breast cancer tissues were obtained from Tianjin Medical University Cancer Institute and Hospital under protocols approved by the ethics committee. Informed consent was obtained from all patients. These specimens were surgically removed from patients with breast cancer from January 2014 to June 2016, and the diagnosis was confirmed through pathological analysis. All pathological and clinical parameters were retrieved from electronic medical records. Immunohistochemical (IHC) staining was performed according to a standard protocol, as described previously^37^. The primary antibody against SHP2 was used at a dilution of 1:150. The expression of SHP2 was calculated by multiplying the percentage and intensity scores. The percentage score was defined as follows: 1 (0%–25%), 2 (26%–50%), 3 (51%–75%), and 4 (76%–100%). The intensity score was defined as follows: 0 (no stained), 1 (moderate staining), and 2 (strong staining). The high-expression group was defined as tissues with a final score greater than 2.

### Tumor xenograft model

Female BALB/c-nude mice (4–5 weeks of age) were purchased from Beijing HFK Bioscience Co., Ltd. All experimental procedures were approved by the Animal Ethical Committee of Tianjin Medical University Cancer Institute and Hospital. For the xenograft model, 5 nude mice in each group were subcutaneously injected with 4 × 10^6^ cells. Tumor sizes were measured and recorded every 4 days, and tumor volumes were calculated with the formula: (length × width^2^)/2. After 32 days, the mice were sacrificed, and tumors were excised, weighed, and photographed. In addition, the expression of SHP2, Cyclin D1, and Ki67 in these tumor sections was examined through IHC staining.

### Statistical analysis

All data are expressed as mean ± SD. The differences among groups were evaluated through one-way or two-way ANOVA in GraphPad Prism 7.00 software (La Jolla, CA, USA). The log-rank test was computed in R to determine the associations between SHP2 expression and the prognosis of patients with breast cancer. Statistical Package for the Social Sciences (SPSS) 13.0 software (SPSS Inc, Chicago, IL, USA) was used to analyze the relationship between SHP2 expression and clinicopathological parameters. *P*-values less than 0.05 were considered statistically significant.

## Results

### Elevated SHP2 expression is associated with poor prognosis in patients with breast cancer

**Figure 1:**
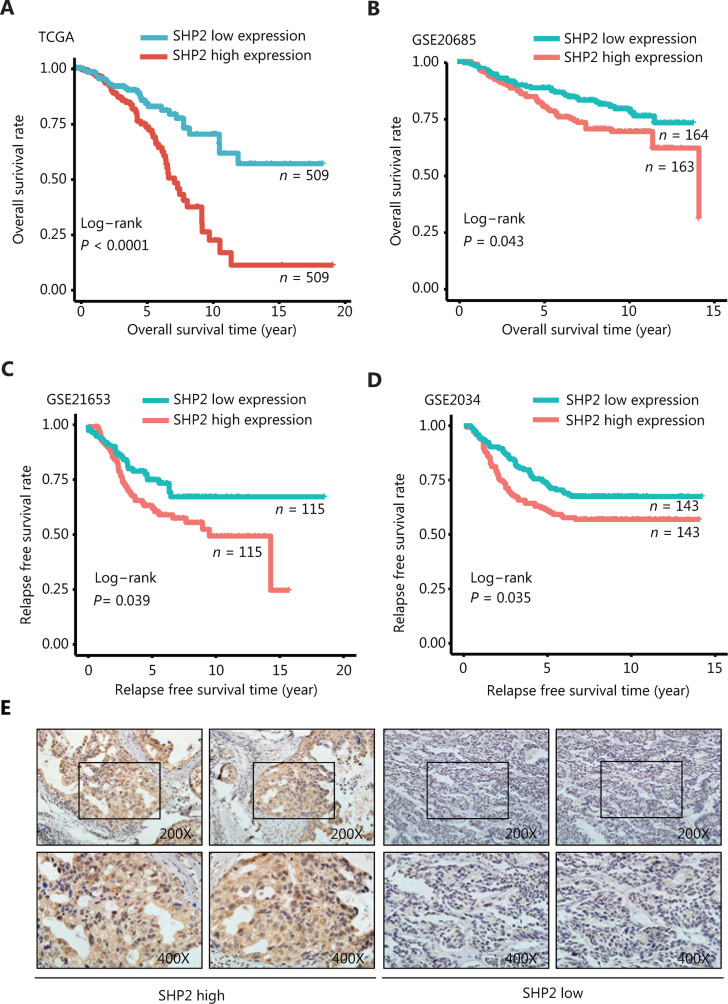
High expression of SHP2 is associated with poor prognosis in patients with breast cancer. (A, B) The overall survival rate in patients with elevated SHP2 expression was significantly poorer than that in patients with low SHP2 expression, on the basis of the TCGA and GSE20685 databases (*P* < 0.001 and *P* = 0.043, respectively). (C, D) The relapse-free survival rate in patients with elevated SHP2 expression was significantly poorer than that in patients with low SHP2 expression, on the basis of the GSE21653 and the GSE2034 databases (*P* = 0.039 and *P* = 0.035, respectively). (E) The expression of SHP2 in breast cancer tissue was detected *via* IHC staining. The expression of SHP2 was scored according to the stained area and staining intensity, and divided into 2 groups with high and low SHP2 expression.

TCGA and the Gene Expression Omnibus (GEO) databases were used to investigate the role of SHP2 in breast cancer and to evaluate the correlation between PTPN11/SHP2 mRNA expression and the prognosis of patients with breast cancer. According to the analysis of the data in TCGA (*n* = 1018), the breast cancer tumors expressing high levels of SHP2 were associated with a markedly poor overall survival rate (**Figure 1A**). In agreement with these data, the overall survival of patients with highly expressed SHP2 was even poorer in the GSE20685 data set (*n* = 327) (**Figure 1B**). Subsequently, the relapse-free survival rate in breast cancer from the GSE21653 (*n* = 230) and the GSE2034 (*n* = 286) data sets were explored. As shown in **Figure 1C** and **1D**, patients with high SHP2 expression had shorter relapse-free survival time than patients with low SHP2 expression. The association between SHP2 expression and breast cancer clinicopathological parameters was investigated through examination of SHP2 expression in 101 breast cancer tissues through IHC staining. As shown in **Figure 1E** and **Table 2**, SHP2 was highly expressed in 40 of the 101 cancer tissue samples. SHP2 was primarily localized in the cytoplasm in breast cancer tissues. Moreover, SHP2 expression was significantly and positively correlated with estrogen receptor expression (*P* = 0.026) (**Table 2**), and the high expression of SHP2 was associated with lymph node metastasis (*P* = 0.039). Strikingly, SHP2 expression was strongly associated with tumor size (*P* = 0.003) and Ki67 levels (*P* = 0.005). Together, these data suggested that the elevated SHP2 expression in breast cancer may be closely associated with cell proliferation, and increased SHP2 levels appear to be associated with the poor prognosis of patients with breast cancer.

### CRISPR/Cas9-mediated PTPN11/SHP2 knockout inhibits proliferation in breast cancer cells

**Figure 2:**
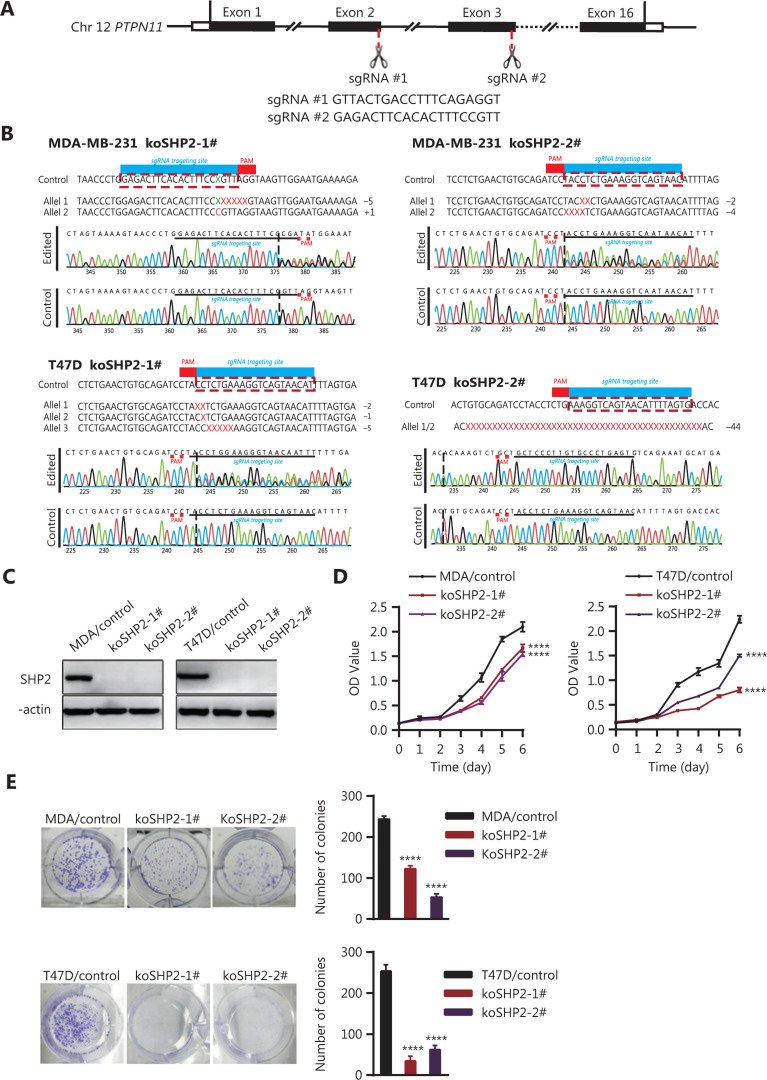
Knockout of SHP2 inhibits the proliferation ability of breast cancer cells. (A) Schematic representation of *PTPN11*-targeting gRNA sequences. Two different guide RNAs were used to target the *PTPN11* gene. (B) Generation of stable SHP2-knockout breast cancer cell lines with a CRISPR/Cas9-mediated gene editing method. DNA sequencing analysis confirmed that the deleted mutations were introduced into the PTPN11 genomic region of the 2 breast cancer cell lines. (C) Western blot analysis of the expression of SHP2 in control and SHP2 knockout cells with β-actin as loading control. (D) CCK8-based assays showed that SHP2 knockout significantly slowed the proliferation rate of the 2 breast cancer cells. Data are presented as mean ± SD, and statistical analysis was carried out with two-way ANOVA (****P < 0.0001). (E) Fewer colonies were observed in the 2 breast cancer SHP2 knockout lines than in the control cells. All data are presented as mean ± SD. Experiments were repeated 3 times. Statistical analysis was carried out with one-way ANOVA (****P < 0.0001).

To explore whether SHP2 regulates breast cancer proliferation, we used CRISPR/Cas9-based gene editing technology to generate SHP2 knockout cells in 2 breast cancer cell lines with 2 different guide RNAs. Multiple monoclonal cells were isolated, and the nucleotide sequences of the target genomic DNA were analyzed. As shown in **Figure 2A** and **B**, DNA sequencing analysis revealed that deletion mutations had been introduced into the target sites in the genomes of the 2 lines of breast cancer cells. These nucleotide deletions resulted in frameshift mutations in the protein coding sequence. Moreover, the results from western blotting indicated that the expression of SHP2 proteins was almost completely eliminated in the SHP2 knockout cell lines (**Figure 2C**). Next, we performed CCK-8 assays to evaluate the cell proliferation ability after SHP2 knockout in the 2 lines of breast cancer cells. As shown in **Figure 2D**, knockout of SHP2 significantly slowed the proliferation rates of the 2 lines of breast cancer cells. This phenomenon was further confirmed by colony formation experiments. SHP2 knockout MDA-MB-231 and T47D cells showed markedly fewer colonies than control cells (**Figure 2E**). Together, these results suggested that SHP2 is required for the proliferation of breast cancer cells.

### Knockout of SHP2 delays G1-to-S phase transition and decreases Cyclin D1 abundance

**Figure 3:**
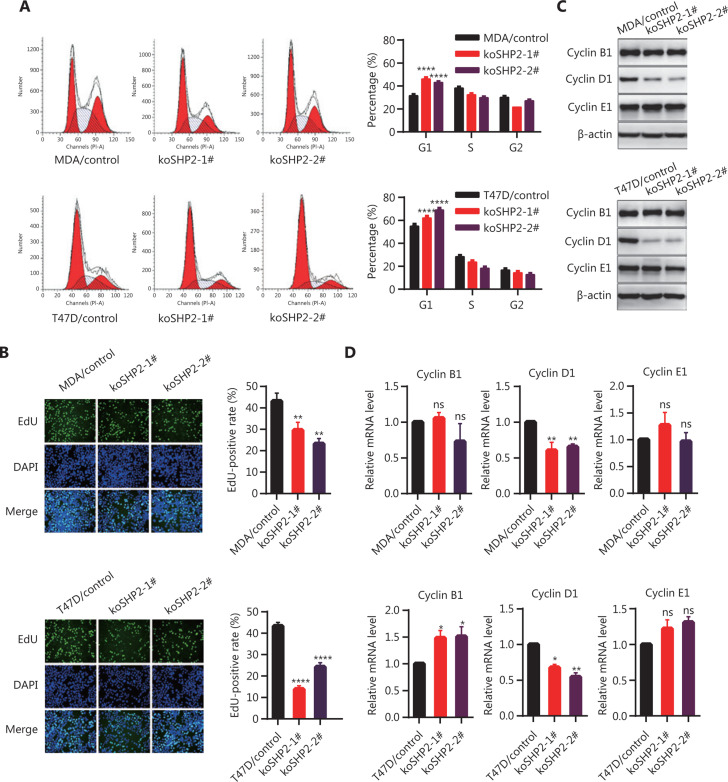
SHP2 knockout delays G1-to-S phase transition through downregulation of Cyclin D1 abundance in breast cancer cells. (A) SHP2 knockout increased the percentage of G1 phase cells and decreased the percentage of G2/S phase cells. The cell cycle was evaluated through flow cytometry assays. The percentages of cells in each cell cycle phase are shown as mean ± SD from 3 independent experiments (****P < 0.0001). (B) EdU incorporation assays showed that the proportion of cells in the S phase was lower in SHP2 knockout cells than control cells. Data are presented as mean ± SD (**P < 0.01, ****P < 0.0001). (C) SHP2 knockout markedly decreased the protein level of Cyclin D1, whereas the expression of Cyclin B1 and Cyclin E1 was not altered. (D) SHP2 knockout decreased the mRNA expression of Cyclin D1. Quantitative PCR analysis of the mRNA expression of Cyclin B1, Cyclin D1, and Cyclin E1 in control and SHP2 knockout cells. Data are presented as mean ± SD. Statistical analysis was carried out with one-way ANOVA (*P < 0.1, **P < 0.01).

To determine whether the decrease in proliferative activity of SHP2 knockout cells might be associated with the obstruction of cell cycle progression, we analyzed the cell cycle phase distribution with flow cytometry assays. The loss of SHP2 inhibited cell cycle progression in breast cancer cells by increasing the proportion of cells in the G1 phase and decreasing the percentage of cells in G2 and the S phases (**Figure 3A**). Moreover, EdU incorporation experiments also confirmed a significantly smaller proportion of cells in S phase in the SHP2 knockout group than in the control group (**Figure 3B**). These data indicated that the loss of SHP2 may delay the G1-to-S phase transition in breast cancer cells. Therefore, we examined the expression of the cell cycle-associated proteins Cyclin B1, Cyclin D1, and Cyclin E1 in the control and SHP2 knockout cells. As shown in **Figure 3C**, the expression of Cyclin D1, a critical regulator of G1-to-S phase transition, was substantially lower in SHP2 knockout cells than control cells. Moreover, our data showed that the phosphorylation of Rb (Ser780), a key downstream molecule of the CDK4–cyclin D1 complex, was significantly lower in SHP2 knockout cells than in control cells (**Supplementary Figure S1**). However, the loss of SHP2 did not affect the abundance of Cyclin E1, another crucial modulator of the G1-to-S phase transition. In addition, expression of the G2/M checkpoint regulator Cyclin B1 was not significantly altered in SHP2 deleted cells. Likewise, quantitative polymerase chain reaction (PCR) analysis showed that the expression of Cyclin D1 mRNA was diminished in SHP2 knockout breast cancer cells, whereas that of Cyclin E1 was unchanged (**Figure 3D**). In addition, the expression of Cyclin B1 mRNA slightly increased after SHP2 deletion in T47D cells, whereas no significant change in the mRNA expression of Cyclin B1 was observed in SHP2-knockout MDA-MB-231 cells (**Figure 3D**). Collectively, these results demonstrated that the knockout of SHP2 delayed the G1-to-S phase transition and decreased Cyclin D1 expression in breast cancer cells.

### SHP2 regulates the protein stability of Cyclin D1 *via* the ubiquitin–proteasome pathway

**Figure 4:**
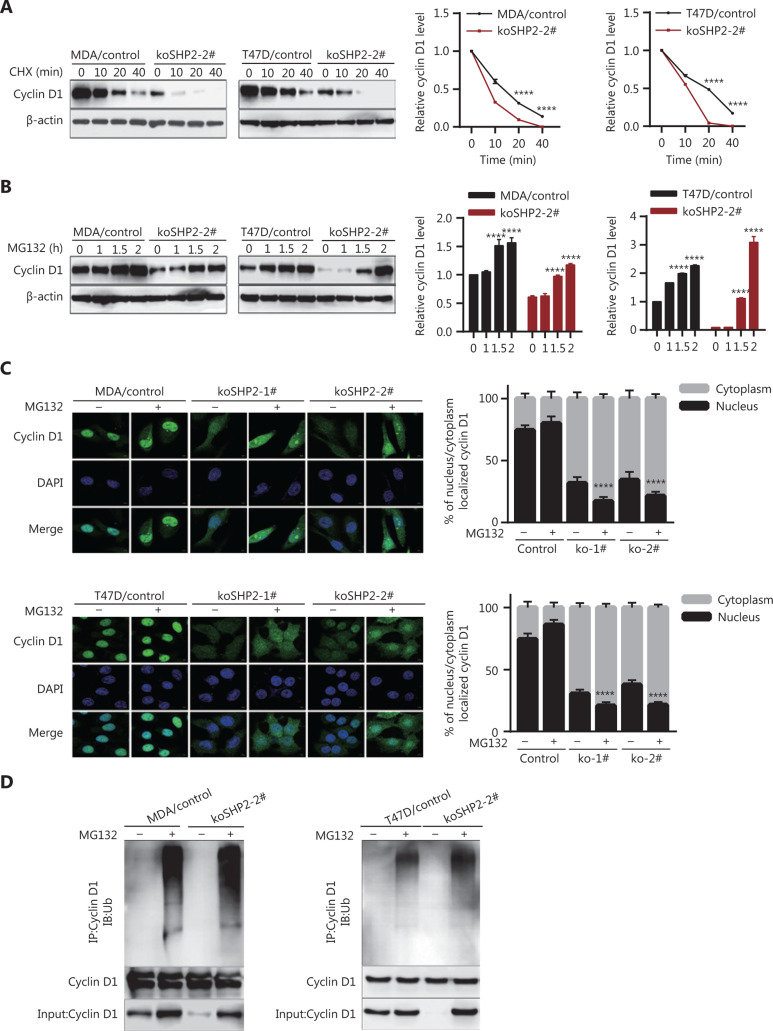
SHP2 knockout promotes Cyclin D1 degradation through the ubiquitin–proteasome pathway. (A) The half-life of Cyclin D1 in SHP2 knockout cells was significantly shorter than that in control cells. The cells were treated with cycloheximide for the indicated times, and the expression of Cyclin D1 was analyzed by western blotting. (****P < 0.0001). (B) The expression level of Cyclin D1 in SHP2 knockout cells was restored by MG132 treatment. The cells were treated with 10 μM of MG132 for the indicated times, and the expression of Cyclin D1 was analyzed by western blotting (****P < 0.0001). (C) Immunofluorescence staining showed that MG132 treatment resulted in an elevation of Cyclin D1 in SHP2 deleted cells. Cyclin D1 was mainly localized in the nuclei in control cells, whereas the increased Cyclin D1 after MG132 treatment in SHP2 deleted cells was mainly localized in the cytoplasm. The quantiﬁcation of Cyclin D1 nucleus/cytoplasm ratio is shown in the right panel (****P < 0.0001). (D) Significantly greater ubiquitinated Cyclin D1 was observed in SHP2 knockout cells than in control cells. The control and SHP2 knockout cells were treated with MG132 or left untreated, and were then lysed and immunoprecipitated with anti-Cyclin D1. The enriched proteins were analyzed by western blotting with anti-Cyclin D1 and anti-ubiquitin antibodies.

To determine whether SHP2 regulates Cyclin D1 protein stability, we treated control and SHP2 knockout cells with CHX to block de novo protein synthesis and then evaluated the rate of Cyclin D1 degradation. Our data clearly demonstrated that the half-life of Cyclin D1 in SHP2 knockout cells was significantly shorter than that in control cells (**Figure 4A**), thus suggesting that the loss of SHP2 accelerated Cyclin D1 degradation. Next, we treated control and SHP2 knockout cells with the proteasome inhibitor MG132 (10 µM). As shown in **Figure 4B**, the decrease in Cyclin D1 caused by SHP2 knockout was restored after MG132 treatment, thus indicating that SHP2 knockout triggered the degradation of Cyclin D1 in breast cancer cells *via* the proteasome-mediated proteolysis pathway (**Figure 4B**). In line with these data, results from immunofluorescence staining showed that the MG132 treatment led to significant increases in Cyclin D1 expression in the control and 2 SHP2-depleted breast cancer cells (**Figure 4C**). Interestingly, Cyclin D1 was mainly localized in the nuclei in control cells in the presence or absence of MG132. In contrast, the increased Cyclin D1 after MG132 treatment in SHP2 knockout cells was mainly distributed in the cytoplasm (**Figure 4C**). These data suggested that SHP2 knockout facilitates nuclear export of Cyclin D1 and its degradation by the ubiquitin–proteasome system. Consistently with these findings, the results of nuclear separation experiments showed that the increased Cyclin D1 protein was mainly located in the cytoplasm in SHP2-deficient cells treated with MG132 (**Supplementary Figure S2**). To further confirm that Cyclin D1 degradation induced by SHP2 loss occurred through the ubiquitin–proteasome pathway, we determined whether the deletion of SHP2 increased ubiquitinated Cyclin D1 in breast cancer cells. These cells were treated with MG132 for 6 h or were left untreated. Cyclin D1 was then immunoprecipitated with anti-Cyclin D1 antibodies and probed with anti-ubiquitin and anti-Cyclin D1 antibodies. As shown in **Figure 4D**, MG132 treatment resulted in significantly greater levels of ubiquitinated Cyclin D1 in SHP2 knockout cells than in control cells. Therefore, these results demonstrated that SHP2 regulates Cyclin D1 protein stability *via* the ubiquitin–proteasome pathway.

### SHP2 knockout-mediated GSK3β activation results in Cyclin D1 threonine 286 phosphorylation and subsequent degradation

**Figure 5:**
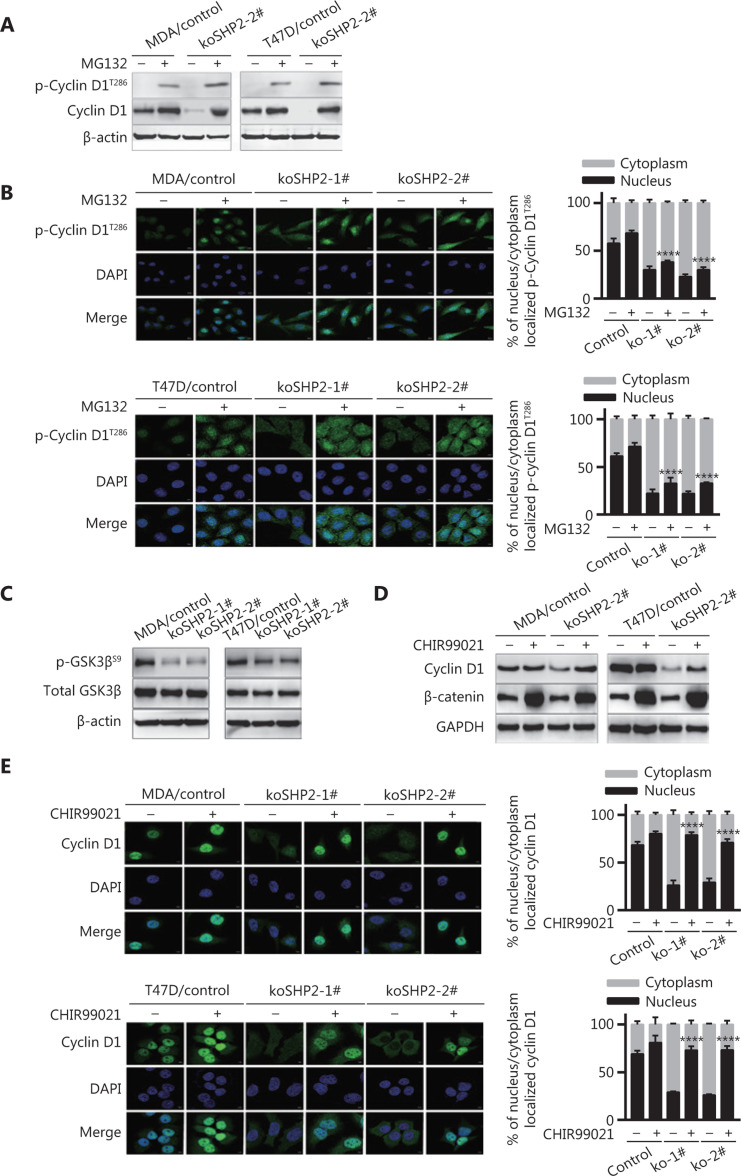
GSK3β-induced T286 phosphorylation of Cyclin D1 is responsible for Cyclin D1 proteasomal degradation. (A) SHP2 knockout increased the protein levels of phosphorylated Cyclin D1 at T286 in the presence of MG132 (10 μM). (B) Immunofluorescence staining showed that MG132 treatment considerably increased the level of phosphorylated Cyclin D1 (T286). Cells were treated with 10 μM of MG132 for 6 h, fixed, and stained with anti-p-Cyclin D1 (T286) antibodies. The quantiﬁcation of the p-Cyclin D1 (T286) nucleus/cytoplasm ratio is shown in the right panel. Statistical analysis was carried out with one-way ANOVA (****P < 0.0001). (C) Western blot analysis of the expression of total and phosphorylated GSK3β (Ser9) in cell lysates from the control and SHP2 knockout cells. (D) The protein level of Cyclin D1 in SHP2 deleted cells recovered after treatment with the GSK3β inhibitor CHIR99021. Control and SHP2 deleted cells were treated with 20 μM of CHIR99021 for 6 h or left untreated, and the expression of Cyclin D1 was analyzed by western blotting. (E) Immunofluorescence staining showed that CHIR99021 treatment significantly increased the nuclear expression of Cyclin D1 in the control and SHP2 deleted cells. The quantification of the Cyclin D1 nucleus/cytoplasm ratio is shown in the right panel (****P < 0.0001).

Cyclin D1 turnover is dependent on threonine 286 (T286) phosphorylation-induced degradation in a ubiquitin-dependent manner^38,39^. Therefore, to investigate whether Cyclin D1 proteolysis induced by SHP2 knockout in breast cancer cells might be associated with T286 phosphorylation, we treated the cells with MG132 and then examined the expression of phosphorylated Cyclin D1. As shown in **Figure 5A**, SHP2 knockout substantially increased p-Cyclin D1 (T286) expression in breast cancer cells when the action of the proteasome was blocked (**Figure 5A**). In addition, the level of phosphorylated Cyclin D1 (T286) was higher in the SHP2 knockout group than in the control group (**Figure 5A**). Consistently with these findings, immunofluorescence staining showed that MG132 treatment significantly elevated the level of p-Cyclin D1 (T286). The p-Cyclin D1 (T286) was mainly distributed in the nuclei of the control cells, whereas most p-Cyclin D1 (T286) was distributed in the cytoplasm in the SHP2 knockout cells. In addition, the fluorescence intensity was higher in SHP2 knockout cells than control cells after addition of MG132 (**Figure 5B**). These data indicated that SHP2 knockout facilitates the nuclear export of p-T286-Cyclin D1 into the cytoplasm. GSK3β is responsible for the phosphorylation of Cyclin D1 on T286^39^. We then investigated the expression of phosphorylated GSK3β at Ser9 in control and SHP2 knockout cells. As shown in **Figure 5C**, the phosphorylation of GSK3β was markedly lower in the 2 SHP2 knockout cell lines than the control cells, thus indicating the activation of GSK3β. To determine whether GSK3β confers a loss of SHP2-mediated Cyclin D1 degradation, we tested the effect of CHIR99021, an inhibitor of GSK3β, on SHP2 knockout-induced Cyclin D1 proteolysis. As shown in **Figure 5D**, CHIR99021 rescued the expression of Cyclin D1 protein caused by SHP2 knockout. In addition, the inhibition of GSK3β by CHIR99021 increased the protein level of β-catenin, a substrate of GSK3β. The phosphorylation of β-catenin by GSK accounted for its degradation. In agreement with these results, immunofluorescence staining showed that CHIR99021 treatment led to a significant increase in the nuclear expression of Cyclin D1 (**Figure 5E**). This result demonstrated that inhibition of GSK3β activity decreased the cytoplasmic translocation of Cyclin D1 from the nuclei in SHP2 knockout cells. Together, these data suggested that SHP2 knockout-mediated GSK3β activation results in T286 phosphorylation and subsequent proteolysis of Cyclin D1.

### SHP2 knockout mediates dephosphorylation and activation of GSK3β through inhibiting the PI3K/AKT signal pathway

**Figure 6:**
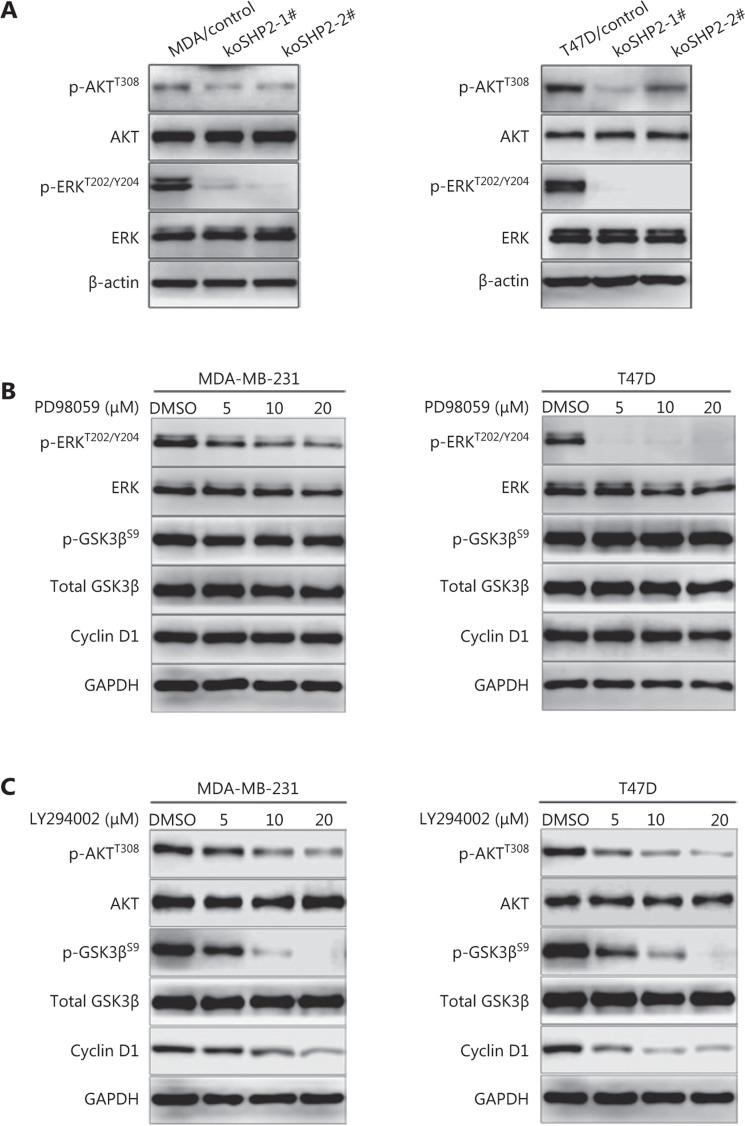
SHP2-deficiency-mediated dephosphorylation and activation of GSK3β through inhibition of the PI3K/AKT signaling pathway. (A) SHP2 knockout decreased the protein levels of phosphorylated AKT (T308) and phosphorylated ERK (T202/Y204) in 2 breast cancer cell lines. (B) Western blot analysis of the levels of Cyclin D1, total and phosphorylated GSK3β (Ser9), and total and phosphorylated ERK (T202/Y204) in 2 breast cancer cell lines pretreated with different concentrations of PD98059 for 6 h. (C) Inhibition of the PI3K/AKT pathway with LY294002 decreased the expression of phosphorylated GSK3β (Ser9) in 2 breast cancer cell lines. The cells were pretreated with different concentrations of LY294002 for 6 h, lysed, and analyzed *via* western blot.

The phosphorylation of GSK3β at Ser9 is mediated through the PI3K/AKT and the MEK/ERK signaling pathways^40,41^. SHP2 is associated with the activation of PI3K/AKT and MEK/ERK signaling in several cell models^42–44^. Indeed, phosphorylation of AKT (T308) and ERK1/2 (T202/Y204) was substantially lower in the 2 SHP2 knockout cell lines than the control cells (**Figure 6A**). To determine which signaling pathway confers phosphorylation of GSK3β in our cell model, we treated breast cancer cells with different concentrations of the ERK inhibitor PD98059. As shown in **Figure 6B**, western blot analysis revealed that PD98059 inhibited ERK1/2 phosphorylation (T202/Y204) in a dose-dependent manner (**Figure 6B**). However, the expression of p-GSK3β (Ser9) and Cyclin D1 was not affected (**Figure 6B**). Next, the effects of LY294002, a PI3K/AKT inhibitor, were further examined on GSK3β (Ser9) phosphorylation and Cyclin D1 expression in the 2 breast cancer cell lines. As shown in **Figure 6C**, the inhibition of PI3K/AKT by LY294002 blocked GSK3β phosphorylation, and the expression of Cyclin D1 protein also significantly decreased in LY294002-treated cells. Collectively, these results suggested that knockout of SHP2 mediated the dephosphorylation and activation of GSK3β through inhibiting the PI3K/AKT signaling pathway.

### Rescued expression of SHP2 restores the cell proliferation ability of breast cancer cells

**Figure 7:**
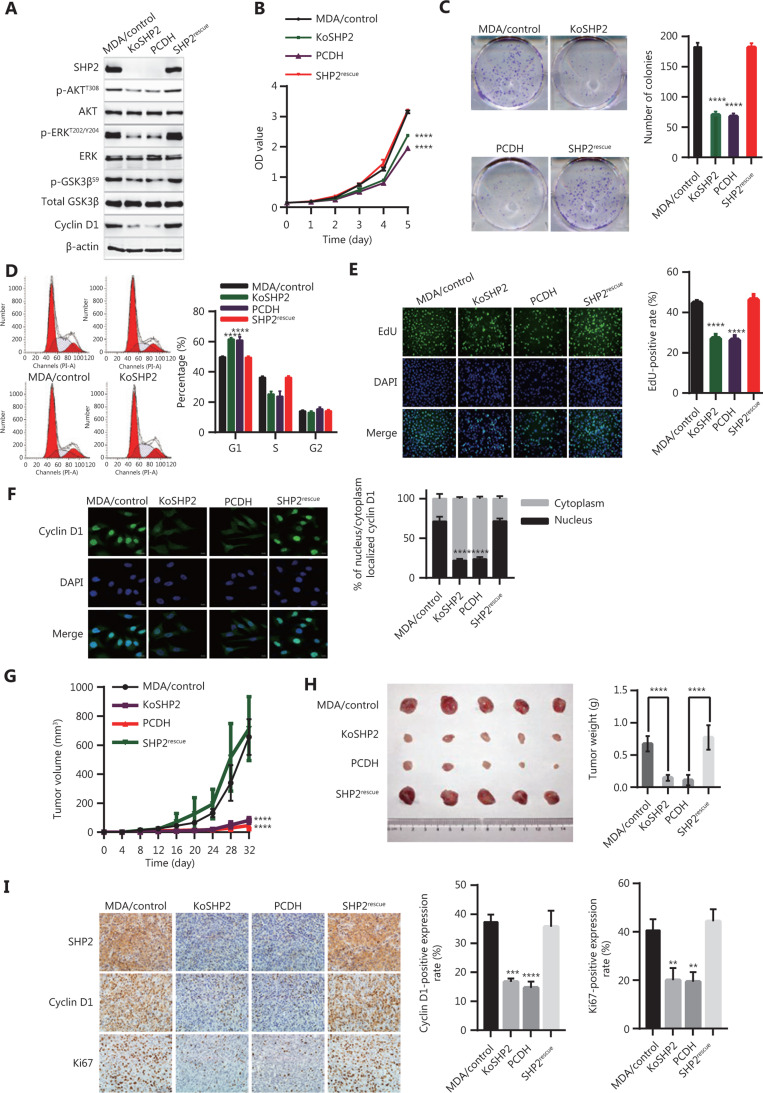
Rescued expression of SHP2 in SHP2 deleted cells restores the proliferation ability of breast cancer cells. (A) Rescued expression of SHP2 restores the expression of phosphorylated AKT (T308), phosphorylated ERK (T202/Y204), phosphorylated GSK3β (Ser9), and Cyclin D1 in SHP2 knockout cells. SHP2 knockout cells were infected with lentivirus for SHP2 expression. The cells were then lysed and analyzed *via* western blotting. (B) CCK-8 assays showed that the re-expression of SHP2 in SHP2 deleted cells restored the cell proliferation ability. Data are shown as mean ± SD. Statistical analysis was performed with two-way ANOVA (****P < 0.0001). (C) Rescued expression of SHP2 restored the colony formation ability of SHP2 knockout cells. Statistical analysis was carried out with one-way ANOVA (****P < 0.0001). (D) Rescued SHP2 expression resulted in a smaller proportion of G1 phase cells than that in SHP2 knockout cells. Data are shown as mean ± SD. Statistical analysis was carried out with one-way ANOVA (****P < 0.0001). (E) EdU incorporation assays showed that the re-expression of SHP2 increased the proportion of cells in S phase. Data are expressed as mean ± SD from 6 independent fields (****P < 0.0001). (F) Rescued SHP2 in SHP2 deleted cells restored the nuclear expression of Cyclin D1. Data are expressed as mean ± SD from 5 independent fields (****P < 0.0001). (G) SHP2 knockout inhibited tumor growth *in vivo*, whereas the re-expression of SHP2 rescued tumor growth defects caused by SHP2 deletion. Statistical analysis was performed with two-way ANOVA (****P < 0.0001). (H) SHP2 knockout in breast cancer cells resulted in a significant decrease in tumor weight, whereas the rescued expression of SHP2 in SHP2 deleted cells resulted in tumors heavier than those in the control group. All data are expressed as mean ± SD (****P < 0.0001). (I) SHP2 knockout decreased the expression of Ki67 and Cyclin D1 in tumor sections, whereas rescued expression of SHP2 restored the expression of these 2 proteins (**P < 0.01, ***P < 0.001, ****P < 0.0001).

According to our findings, SHP2 knockout inhibited cell proliferation *via* suppression of the AKT/GSK3β/Cyclin D1 signaling pathway. Therefore, we reasoned that SHP2 restoration might result in regained proliferative capacity in SHP2 knockout cells. We tested this hypothesis by infecting the SHP2 knockout cells with lentivirus for expression of SHP2. As shown in **Figure 7A**, the SHP2 expression in SHP2 knockout cells was successfully restored, whereas the cells infected with control virus (PCDH) did not show rescued SHP2 expression. Moreover, the expression of p-AKT (T308), p-ERK (T202/Y204), p-GSK3β (Ser9), and Cyclin D1 in SHP2-restored cells was effectively rescued, in contrast to the results in control virus-expressing cells. Next, we compared the cell proliferation ability among these cells. As shown in **Figure 7B**, CCK-8 assays indicated that the re-expression of SHP2 significantly rescued cell proliferation ability to levels similar to those of wild-type cells, whereas the control virus did not ameliorate the cell proliferation defects. In agreement with the above results, colony formation assays showed that rescue with SHP2 resulted in recovery of cell proliferation ability (**Figure 7C**). We next examined the cell cycle with flow cytometry and found that SHP2 rescue decreased the proportion of cells in G1 phase and increased the percentage of cells in S phase (**Figure 7D**). In agreement with these results, EdU incorporation assays showed that the re-expression of SHP2 increased the proportion of S-phase cells (**Figure 7E**). Moreover, the expression and nuclear localization of Cyclin D1 were restored after SHP2 re-expression (**Figure 7F**). We additionally determined the effect of SHP2 expression on the proliferative potential of breast cancer cells in an *in vivo* nude mouse model through subcutaneous injection of control, KoSHP2, KoSHP2^PCDH^, and SHP2-rescued cells. The growth rates of the corresponding tumors were measured within 32 days. As shown in **Figure 7G**, the tumors from SHP2 knockout cells grew significantly more slowly than those from control cells, and the growth rates of tumors from SHP2-rescued cells were similar to those of tumors from control cells. These results were consistent with our *in vitro* cell proliferation results. In line with these data, SHP2 knockout in breast cancer cells resulted significantly decreased the tumor weight (**Figure 7H**). We also examined SHP2, Ki67, and Cyclin D1 expression in xenografted tumors. As shown in **Figure 7I**, IHC staining of Ki67 and Cyclin D1 was significantly downregulated after SHP2 deletion. Together, these results suggested that SHP2 is required for the *in vitro* proliferation of breast cancer cells and *in vivo* tumor growth.

## Discussion

Recent studies have linked the tyrosine phosphatase SHP2 to malignant behavior of tumor cells and poor prognosis of patients with various carcinomas^6,32,33,45,46^. Nevertheless, the precise mechanism through which SHP2 promotes breast cancer progression was largely undefined. In the present study, we demonstrated that SHP2 functions as a key modulator of the proliferation of breast cancer cells by promoting the G1-to-S phase transition through regulating Cyclin D1 stability *via* the PI3K/AKT/GSK3β signaling pathway. CRISPR/Cas9-mediated knockout of SHP2 decreased Cyclin D1 protein abundance and consequently resulted in cell cycle defects and diminished cell proliferation *in vitro* and tumor growth *in vivo*. In contrast, overexpression of SHP2 increased the Cyclin D1 abundance and accelerated the cell cycle by promoting the transition from G1 phase to S phase (**Supplementary Figure S3**). Moreover, the rapid proteolysis of Cyclin D1 induced by SHP2 loss was mainly due to proteasome-dependent degradation. Our results indicated that the deletion of SHP2 decreased PI3K/AKT signaling, thereby resulting in GSK3β’s dephosphorylation and subsequent activation. GSK3β-mediated phosphorylation of Cyclin D1 at T286 then promoted rapid degradation of Cyclin D1 in SHP2 knockout cells. Conversely, the restoration of SHP2 reversed the Cyclin D1 decrease and cell cycle defects, and rescued the cell proliferation arrest *in vitro* and the tumor growth inhibition *in vivo* caused by SHP2 knockout. Our study thus uncovered the mechanism through which SHP2 regulates breast cancer proliferation.

Although SHP2 has been widely recognized as an oncogenic driver in a variety of malignancies, SHP2 can also function as a tumor suppressor in hepatocellular carcinoma, esophageal squamous cell cancer, and cartilage tumors^23–25,47^. To confirm the function of SHP2 in breast cancer, we knocked out the *SHP2* gene in 2 breast cancer cell lines with CRISPR/Cas9-based genome editing. SHP2 loss significantly induced proliferation arrest *in vitro* and tumor growth inhibition *in vivo*, whereas rescuing the expression of SHP2 in SHP2 knockout cells reversed the proliferation defect. Hence, these data supported the tumor-promoting function of SHP2 in breast cancer. These results were also consistent with previous findings demonstrating that SHP2 is a key regulator of cell proliferation in prostate cancer^33^. In addition, the elevated SHP2 expression in breast cancer tissues was positively correlated with tumor size and strong staining for Ki67, a proliferation marker. This result suggested the pro-oncogenic potential of SHP2 in breast cancer. Moreover, SHP2 knockout slowed cell cycle progression by inhibiting the G1-to-S phase transition. In agreement with our data, a previous study has demonstrated that silencing SHP2 with shRNA inhibits the proliferation of prostate cancer cells by arresting the cell cycle at G1 phase^33^. In summary, SHP2 regulates cell proliferation through promoting cell cycle progression. To our knowledge, this study is the first to report the use of CRISPR/Cas9-based SHP2 gene knockout in breast cancer cells.

The G1-to-S phase transition is regulated by Cyclin D1 and Cyclin E1^48,49^. Herein, the loss of SHP2 induced a significant decrease in Cyclin D1 expression at the mRNA and the protein levels, whereas SHP2 knockout did not affect the expression of Cyclin E1. In addition, the expression of Cyclin B1, a G2/M phase specific regulator, was not altered in SHP2 knockout cells. These results indicated that SHP2 loss inhibits cell cycle progression *via* the downregulation of Cyclin D1. Cyclin D1 is an unstable and short-lived protein whose stability is mainly modulated by a ubiquitination-mediated and proteasome-dependent pathway^49,50^. In the present study, the inhibition of de novo protein synthesis by CHX notably shortened the half-life of Cyclin D1 in the 2 SHP2 knockout breast cancer cell lines, thus indicating that SHP2 loss resulted in rapid degradation of Cyclin D1. In contrast, blocking proteasome activity with MG132 significantly rescued the decrease in Cyclin D1 protein caused by SHP2 knockout. This result further confirmed that SHP2 deletion promotes Cyclin D1 instability and proteolysis. Moreover, after 2 h of MG132 treatment, the increase in Cyclin D1 protein levels in 2 SHP2 knockout cell lines was close to the endogenous levels in control cells. Given that the decrease in Cyclin D1 mRNA in SHP2 knockout cells was not as clear as the decrease in its protein level, lower Cyclin D1 stability due to SHP2 knockout may play a more important role in decreasing Cyclin D1 abundance rather than cellular transcription. Furthermore, most Cyclin D1 was localized in the nuclei in control cells before or after MG132 treatment, whereas SHP2 loss induced a marked increase in cytoplasmic Cyclin D1 in the presence of MG132. In contrast, the re-expression of SHP2 in SHP2 knockout cells restored nuclear Cyclin D1 expression. These data demonstrated that SHP2 is required for the nuclear localization of Cyclin D1. Subcellular localization is well known to regulate the ubiquitination and subsequent proteolysis of Cyclin D1^38,39^. As expected, the level of ubiquitinated Cyclin D1 in SHP2 knockout cells was clearly higher than that in control cells. Hence, the knockout of SHP2, which prevented Cyclin D1 from localizing to the nucleus and promoted its cytoplasmic translocation, facilitated the degradation of Cyclin D1 by the ubiquitin–proteasome system.

Cyclin D1 must be phosphorylated at T286 to be degraded^38^. In the present study, the blockage of proteasome activity by MG132 increased the level of phosphorylated Cyclin D1 in breast cancer cells. Interestingly, the level of p-T286–Cyclin D1 in SHP2 knockout cells was substantially higher than that in control cells, thereby indicating that the SHP2 loss increased Cyclin D1 phosphorylation. T286 phosphorylation of Cyclin D1 facilitates its translocation from the nucleus to the cytoplasm and its rapid proteolysis^38,39^. In line with this finding, our data showed that p-T286-Cyclin D1 was localized mainly in the cytoplasm in the 2 SHP2 knockout cell lines. GSK3β is a well-known kinase that phosphorylates Cyclin D1 on T286, thus inducing its degradation^38,39^. In the present study, the expression of phosphorylated GSK3β at Ser9 was markedly lower in the 2 SHP2 knockout cell lines than in control cells. This result indicated an increase in this kinase’s activity. Hence, GSK3β-mediated Cyclin D1 phosphorylation may be responsible for the rapid proteolysis of Cyclin D1 caused by SHP2 loss. In support of this hypothesis, blocking GSK3β activity with CHIR99021 substantially inhibited the decrease in Cyclin D1 abundance induced by SHP2 knockout. Moreover, GSK3β inhibition resulted in a significant increase in the nuclear expression of Cyclin D1 in SHP2 knockout breast cancer cells. Collectively, these findings suggested that the SHP2 knockout-mediated dephosphorylation and activation of GSK3β confer the T286 phosphorylation and the subsequent cytoplasmic translocation and degradation of Cyclin D1.

The mechanism through which SHP2 regulates GSK3β phosphorylation at Ser9 warrants further investigation. The best-characterized mechanism underlying the phosphorylation of GSK3β at Ser9 is regulated by the protein kinase AKT^41^. ERK1/2 has also been reported to regulate p-Ser9–GSK3β in several cell models^40^. In addition, SHP2 has been linked to the activation of the RAS/MEK/ERK and PI3K/AKT pathways^42–44^. Here, SHP2 loss considerably decreased the phosphorylation of AKT (T308) and ERK1/2 (T202/Y204) in 2 breast cancer cell lines, thus indicating the inactivation of these 2 signaling pathways. Therefore, the decrease in the p-Ser9–GSK3β in SHP2 knockout cells may be attributable to the decline in the phosphorylation of AKT (T308) and/or ERK1/2 (T202/Y204). As expected, blocking PI3K/AKT signaling with LY294002 substantially decreased the expression of phosphorylated AKT (T308) and GSK3β (Ser9) and decreased the abundance of Cyclin D1. Conversely, the re-expression of SHP2 in SHP2 knockout cells restored AKT and GSK3β phosphorylation and the abundance of Cyclin D1. However, the suppression of ERK1/2 phosphorylation by PD98059 in the 2 breast cancer cell lines had no clear effects on the expression of p-Ser9–GSK3β and the abundance of Cyclin D1. Thus, ERK1/2 signaling did not regulate the phosphorylation of GSK3β on Ser9. Together, these results suggested that AKT inactivation due to SHP2 loss confers dephosphorylation and activation of GSK3β and subsequently phosphorylates and accelerates the degradation of Cyclin D1. However, given that PI3K/AKT signaling is a key pathway regulating cell proliferation, the effects of other protein alterations caused by PI3K/AKT inactivation in SHP2 knockout cells on cell proliferation cannot be completely excluded, and the detailed mechanism remains to be further investigated.

## Conclusions

**Figure 8:**
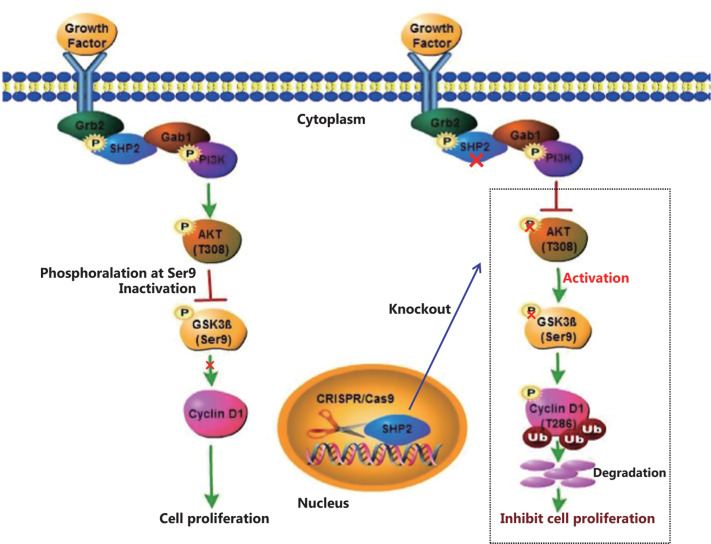
A proposed schematic model: CRISPR/Cas9-mediated knockout of SHP2 inhibits breast cancer proliferation by regulating Cyclin D1 stability *via* the PI3K/AKT/GSK3β signaling pathway.

In summary, our study demonstrates that SHP2 has an oncogenic function in breast cancer. SHP2 is required for cell proliferation *via* regulating Cyclin D1 stability and promoting cell cycle progression. SHP2 knockout attenuates the activation of PI3K/AKT signaling and causes the dephosphorylation and resultant activation of GSK3β. Activated GSK3β phosphorylates Cyclin D1 and promotes Cyclin D1 translocation from the nucleus to the cytoplasm, thereby facilitating its degradation through the ubiquitin–proteasome system. Our study uncovered the mechanism through which SHP2 regulates breast cancer proliferation. Consequently, SHP2 may function as a potential therapeutic target for breast cancer (**Figure 8**).

## Figures and Tables

**Table 1:** Primers used in this study

Name	Primer	Sequence	Length (bp)
Cyclin B1	Upper	5′ CAGGAGACCATGTACATGAC 3′	199
	Lower	5′ CCATCTGTCTGATTTGGTGG 3′	
Cyclin D1	Upper	5′ TGCATCTACACCGACAACTCC 3′	172
	Lower	5′ CGTGTTTGCGGATGATCTGTT 3′	
Cyclin E1	Upper	5′ GGAGATGAAATTCTCACCATGG 3′	195
	Lower	5′ CAGGACACAGAGATCCAACAG 3′	
β-actin	Upper	5′ GGGACCGTAGCGCCTGCGACT 3′	148
	Lower	5′ TCGTCCATGGCCCGCTGACTC 3′	

**Table 2:** Associations between SHP2 expression and clinicopathological parameters

Variables	*n*	*n*		*P*
		SHP2 low	SHP2 high	
Age (years)			0.415
< 50	47	26	21
≥ 50	54	35	19
T			0.003*
T1	65	46	19
T2	32	15	17
> T2	4	0	4
N			0.039*
N0	72	50	22
N1/N2	21	9	12
ER			0.026*
Negative	32	25	7
Positive	65	35	30
PR			0.834
Negative	46	28	18
Positive	51	33	18
HER-2			0.219
Negative	23	12	11
Positive	70	47	23
Ki67 (%)			0.005*
≤ 30%	46	35	11
> 30%	44	20	24
P53			0.057
Negative	36	28	8
Positive	27	14	13
Histological grade			0.202
1	8	7	1
2	54	32	22
3	9	7	2

*P < 0.05 was considered statistically significant.
