# Supplementary material for: SHP2 promotes proliferation of breast cancer cells through regulating Cyclin D1 stability via the PI3K/AKT/GSK3β signaling pathway
Source: Cancer Biol Med. 2020 Aug 15;17(3):707–25. doi: 10.20892/j.issn.2095-3941.2020.0056 (PMC7476086; doi:10.20892/j.issn.2095-3941.2020.0056)
Supplement: Supplementary file 1 [file cbm-17-707-s001.pdf]

Supplementary materials

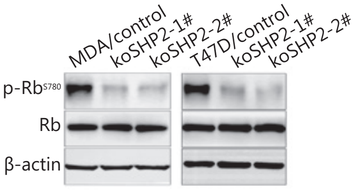

Figure S1 Western blot assays revealed that the p-Rb (Ser780) protein levels were markedly lower in the SHP2 knockout cells than the control cells.

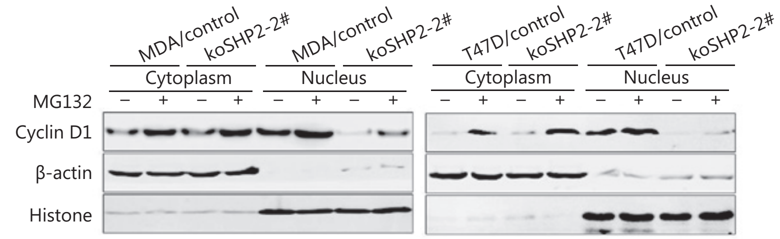

Figure S2 Nuclear extraction assays showed that Cyclin D1 was mainly distributed in the nuclei in control cells, while the elevated Cyclin D1 after MG132 treatment in SHP2 knockout cells were mainly distributed in the cytoplasm.

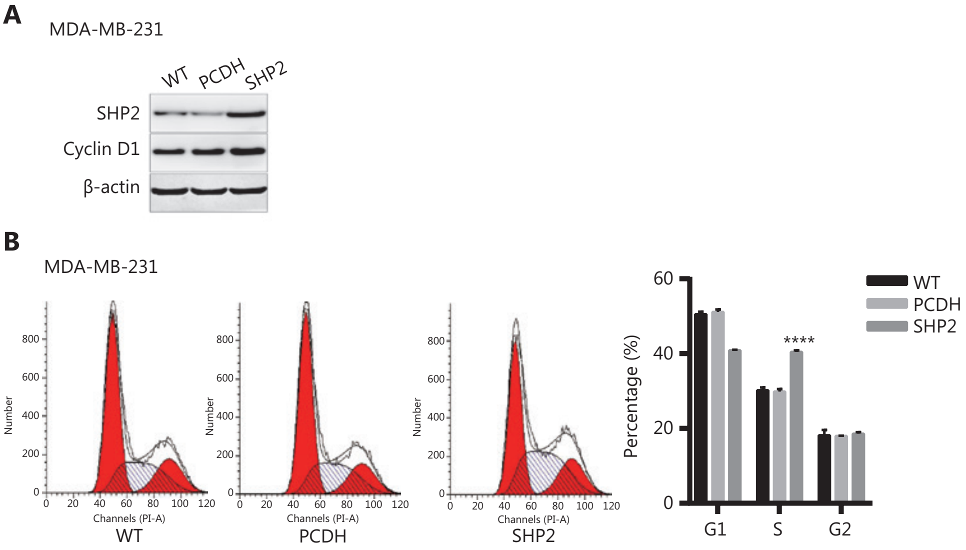

Figure S3 Overexpression of SHP2 promoted the cell cycle progression. (A) Western blot assays showed that the infection of pCDH-SHP2 increased the expression of Cyclin D1. (B) Flow cytometry-based cell cycle assays showed that overexpression of SHP2 decreased the percentage of cells in the G1 phase and increased the proportion of cells in the S phase. The percentage of cells in each cell cycle phase are shown as mean ± SD from 3 independent studies (*****P* < 0.0001).

Supplementray materials and methods

Nuclear extraction

The cells were harvested in ice-cold subcellular fractionation buffer immediately. Then passed the lysate through a needle 10 times using a 1 mL syringe and leaved on ice for 20 min. Followed by 10,000 × g centrifugation and collected the supernatant as cytoplasmic fraction. The precipitate was washed with the buffer and used the needle three times as above, and centrifuged at 14,000 × g for 10 min to obtain nuclear fraction. Protein concentrations were quantitated, and followed by western blotting analysis. Histone (RM2005, Ray Antibody, Beijing, China) and β-actin served as internal controls for nuclear and cytoplasmic compartments, respectively.
